# Improvement of Cognitive Function in Ovariectomized Rats by Human Neural Stem Cells Overexpressing Choline Acetyltransferase via Secretion of NGF and BDNF

**DOI:** 10.3390/ijms23105560

**Published:** 2022-05-16

**Authors:** Eun-Jung Yoon, Yunseo Choi, Dongsun Park

**Affiliations:** 1 Department of Biology Education, Korea National University of Education, Cheongju 28173, Korea; 315836@naver.com (E.-J.Y.); yunseo0921@naver.com (Y.C.); 2 Department of Counseling, Health, and Kinesiology, College of Education and Human Development, Texas A&M University-San Antonio, One University Way, San Antonio, TX 78224, USA

**Keywords:** neurotrophin, estradiol, cholinergic system, memory, ovariectomy, neural stem cells

## Abstract

Menopause is associated with memory deficits attributed to reduced serum estrogen levels. We evaluated whether an increase in brain-derived neurotrophic factor (BDNF) and nerve-growth factor (NGF) levels, through transplantation of choline acetyltransferase (ChAT)-overexpressing neural stem cells (F3.ChAT), improved learning and memory in ovariectomized rats. PD13 mouse neuronal primary culture cells were treated with estradiol or co-cultured with F3.ChAT cells; choline transporter1 (CHT1), ChAT, and vesicular acetylcholine transporter (VAChT) expression was evaluated using real-time PCR. The relationship between estrogen receptors (ERs) and neurotrophin family members was analyzed using immunohistochemistry. After the transplantation of F3.ChAT cells into OVx rats, we evaluated the memory, ACh level, and the expression of ER, neurotrophin family proteins, and cholinergic system. Estradiol upregulated CHT1, ChAT, and VAChT expression in ER; they were co-localized with BDNF, NGF, and TrkB. Co-culture with F3.ChAT upregulated CHT1, ChAT, and VAChT by activating the neurotrophin signalling pathway. Transplantation of F3.ChAT cells in OVX animals increased the ACh level in the CSF and improved memory deficit. In addition, it increased the expression of ERs, neurotrophin signaling, and the cholinergic system in the brains of OVX animals. Therefore, the estradiol deficiency induced memory loss by the down-regulation of the neurotrophin family and F3.ChAT could ameliorate the cognitive impairment owing to the loss or reduction of estradiol.

## 1. Introduction 

Estrogen plays an important role in physiology and metabolism in women and estrogen deficiency induces various diseases and abnormalities, with cognitive dysfunction and memory disorders being the most common [1,2,3]. Estrogen influences neuronal connectivity through the formation of new synaptic connections in the brain and cognitive function by increasing the activity of the cholinergic system [4,5]. Estrogen deficiency induces memory impairment in ovariectomized animals [6]. In addition, estrogen upregulates the expression of neurotrophin family proteins, such as brain-derived neurotrophic factor (BDNF), in the hippocampus [7]. Estrogen influences memory-related processes and prevents a decline in neuronal function [8].

Neurotrophins are a family of proteins that regulate neural survival, development, function, and plasticity [9,10,11,12]. Nerve growth factor (NGF) is a member of the neurotrophin family that supports neural survival after brain damage [10]. BDNF supports neural survival, growth, and synaptic plasticity in the hippocampus and the cortex [9]. NGF and BDNF influence memory function and the increase in NGF and BDNF levels help recover learning and memory in Alzheimer’s animal models [11,12]. In addition, memory deficits can be restored by NGF and BDNF through the transplantation of stem cells [13,14,15].

Acetylcholine (ACh) is a key neurotransmitter in the memory process [16]. Both NGF and BDNF influence cholinergic neurons and increase ACh concentrations [17,18,19]. In addition, estrogen facilitates cholinergic neurotransmission by increasing the activity and mRNA levels of choline acetyltransferase (ChAT) [20]. Therefore, in menopausal women, estrogen replacement therapy increases cognitive function, and upregulation of NGF and BDNF improves cognitive function and menopausal symptoms [21,22].

In an earlier study, we developed neural stem cells overexpressing ChAT (F3.ChAT). F3.ChAT cells restore cognitive function via acetylcholine synthesis and amyloid beta elimination [23]. In addition, resistance exercises enhanced cognitive function in ovariectomized rats via the upregulation of BDNF and NGF mRNA expression [6,24,25,26]. Therefore, in this study, we aimed to demonstrate that an increase in the NGF and BDNF levels in the brain improves learning and memory function in ovariectomized rats using stem cells. In addition, we aimed to elucidate their signaling pathways by evaluating the relationship between estrogen receptors and the neurotrophin family.

## 2. Results

### 2.1. Treatment with Estradiol Upregulates the Cholinergic System

**Figure 1:**
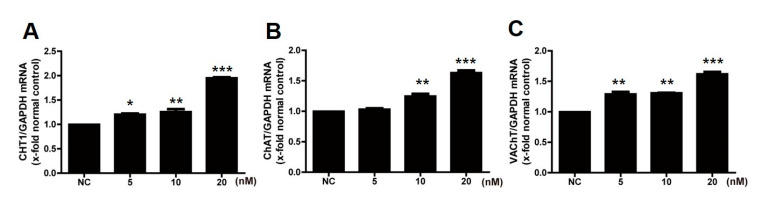
Expression of CHT1 (**A**), ChAT (**B**), and VAChT (**C**) in primary forebrain neurons after treatment with estradiol. Gene expression was analyzed by real-time PCR. CHT1; choline transporter1, ChAT; choline acetyltransferase, VAChT; vesicular acetylcholine transporter. * Significantly different from normal control (*p* < 0.05). ** Significantly different from normal control (*p* < 0.005). *** Significantly different from normal control (*p* < 0.001).

To investigate the effects of estradiol on the cholinergic system, mouse primary neuronal cells were treated with estradiol. Treatment with estradiol (0, 5, 10, and 20 nm) significantly increased the expression of cholinergic systems, such as choline transporter (CHT), ChAT, and vesicular acetylcholine transporter (VAChT) (Figure 1), in a dose-dependent manner.

### 2.2. Estrogen Receptors Co-Localize with Neurotrophin Family

**Figure 2:**
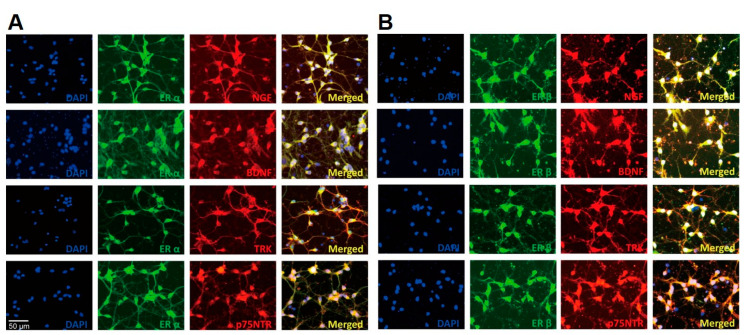
Localization of estrogen receptor (ER) α (**A**) and β (**B**) with NGF, BDNF, TRK, and p75NTR in primary forebrain neuron.

To check the localization of estrogen receptors and neurotrophin family proteins, a double immunocytochemistry analysis was performed with estrogen receptor alpha (ERα) and beta (ERβ) and the neurotrophin family members, NGF, BDNF, TRK, and p75NTR (Figure 2). Both ERα and ERβ were co-localized with neurotrophin family proteins.

The localization was analyzed using immunocytochemistry. ER α and ERβ were stained with Alexa Fluor 488 (green), and the neurotrophin family proteins were stained with Alexa Fluor 594 (red). The cells were counterstained with 4,6-diamino-2-phenylindole (DAPI) to confirm cellular nuclei. NGF, nerve growth factor; BDNF, brain-derived neurotrophic factor; TRK, tyrosine receptor kinase; p75NTR, p75 neurotrophin receptor. Scale bar = 50 μm, magnification ×200.

### 2.3. F3.ChAT Cells Release NGF and BDNF

**Figure 3:**
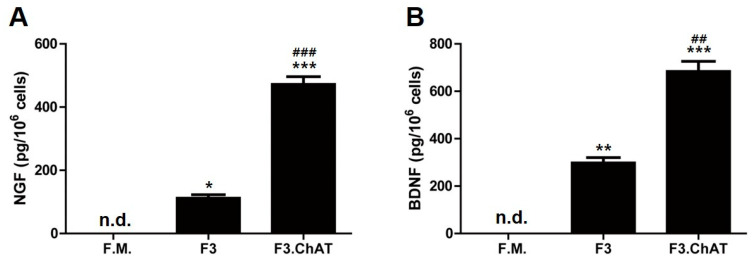
Concentration of NGF (**A**) and BDNF (**B**) in F3 and F3.ChAT cells cultured in conditioned medium. The levels of NGF and BDNF were analyzed using an ELISA kit. The experiments were performed in triplicate, and mean values are presented. F.M.; Fresh mediumm, n.d.; not detected. * Significantly different from the fresh medium (*p* < 0.05). ** Significantly different from the fresh medium (*p* < 0.005). *** Significantly different from the fresh medium (*p* < 0.001). ## Significantly different from the F3 (*p* < 0.005). ### Significantly different from the F3 (*p* < 0.001).

To investigate whether NGF and BDNF increased the levels of cholinergic markers, F3 and F3.ChAT cells were used (Figure 3). To confirm the secretion of NGF and BDNF from F3 and F3.ChAT cells, their concentrations were measured in a conditioned medium. Both F3 and F3.ChAT release NGF and BDNF (Figure 3). The secretion of NGF and BDNF from F3.ChAT cells was higher than that from F3 cells.

### 2.4. Co-Culture with F3.ChAT Cells Enhances Neurotrophin Signaling Pathway

**Figure 4:**
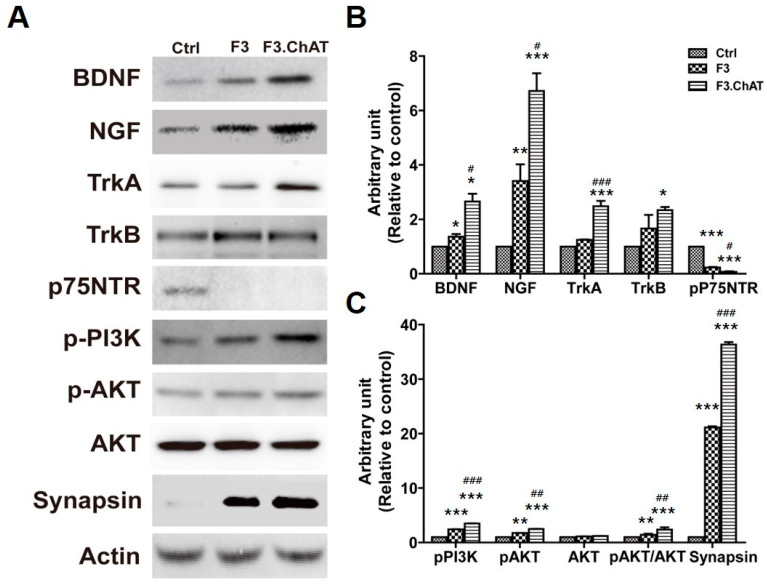
Expression of proteins related to the neurotrophin pathway. (**A**) Representative bands of protein related with the neurotrophin pathway. (**B**,**C**) The band densities were normalized to that of actin. * Significantly different from the normal control (*p* < 0.05). ** Significantly different from the normal control (*p* < 0.005). *** Significantly different from the normal control (*p* < 0.001). # Significantly different from the F3 (*p* < 0.05). ## Significantly different from the F3 (*p* < 0.005). ### Significantly different from the F3 (*p* < 0.001).

F3 or F3.ChAT cells were co-cultured with primary cells in a transwell (pore 0.4 μm); the levels of the neurotrophin signaling pathway molecules were analyzed (Figure 4). The expression of NGF and BDNF in primary cells co-cultured with F3 or F3.ChAT cells was higher than that in the normal control. In addition, the receptors, Pan-Trk and TrkB, were upregulated; however, the expression of p75NTR was downregulated. This resulted in increased PI3k and AKT expression involved in the neurotrophin pathway, which induces cell proliferation [27,28]. Interestingly, synapsin, which influences synaptic plasticity, was upregulated. The expression of proteins in F3.ChAT-co-cultured cells was higher than that in the F3-co-cultured cells.

### 2.5. Co-Culture with F3.ChAT Cells Upregulates the Cholinergic System

**Figure 5:**
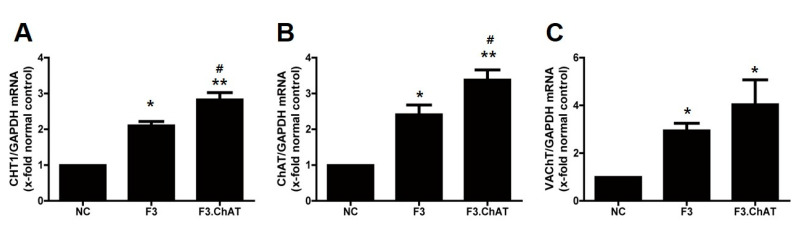
Expression of CHT1 (**A**), ChAT (**B**), and VAChT (**C**) in primary forebrain neurons co-cultured with F3 and F3.ChAT cells. The F3 and F3.ChAT cells were seeded at 1 × 10^5^ cells per ml in the upper chambers of 6-well transwell plates (pore 0.4 μm) on the primary forebrain neuron. After a 24 h incubation, the cells were used for performing real time PCR. * Significantly different from normal control (*p* < 0.05). ** Significantly different from the normal control (*p* < 0.005). # Significantly different from the F3 (*p* < 0.05).

In parallel to that of the neurotrophin signaling pathway, expression of CHT1, ChAT, and VAChT was significantly enhanced by co-culture with F3 and F3.ChAT cells, compared to that in the normal controls (Figure 5). Their expression in primary cells co-cultured with F3.ChAT cells were higher than that in the cells co-cultured with F3 cells.

### 2.6. Transplantation of F3.ChAT Cells Improves Memory Deficit in Ovariectomized Rat

**Figure 6:**
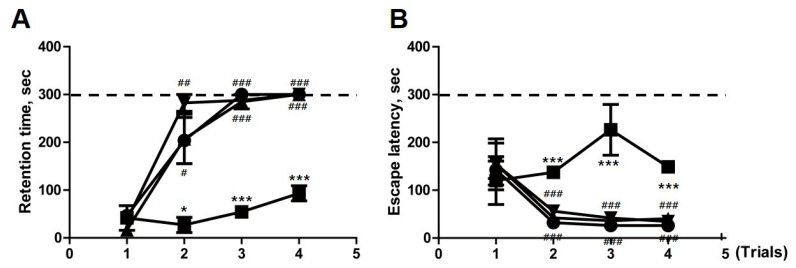
Cognitive function of ovariectomized rats transplanted with F3 or F3.ChAT cells. Cognitive function was analyzed through passive avoidance performance (**A**) and Morri water-maze performance (**B**). The endopind was set to 300 s, if the animals stayed in light chamber in passive avoidance or failed to find the platform in Morris water-maze performance. ●; normal group, ■; Ovariectomy (OVx) group, ▲; OVx + F3 cells group, ▼; OVx + F3.ChAT cells group. * Significantly different from the normal control (*p* < 0.05). *** Significantly different from the normal control (*p* < 0.001). # Significantly different from the OVx (*p* < 0.05) group. ## Significantly different from the OVx (*p* < 0.05) group. ### Significantly different from the OVx (*p* < 0.05) group.

After confirming the in vitro effects of NGF and BDNF from the co-culture of F3 and F3.ChAT cells with primary cells, the in vivo cognitive improvement effect of F3 and F3.ChAT were investigated in an ovariectomized animal model. Three weeks after ovariectomy, animals were subjected to a passive avoidance test and water-maze tests to confirm memory deficits (data not shown). F3 and F3.ChAT cells were transplanted into the ovariectomized rats via the tail vein. Four weeks after the F3 and F3.ChAT transplantations, the rats in the OVx group (control) displayed profound impairments in memory acquisition and retention in both the passive avoidance test and water maze performance (Figure 6). However, the transplantation of F3 and F3.ChAT cells significantly improved memory acquisition in the passive avoidance test. Similar improvements in memory acquisition and retention were observed in the water-maze performance.

### 2.7. Transplantation of F3.ChAT Cells Restores ACh Concentration in CSF

**Figure 7:**
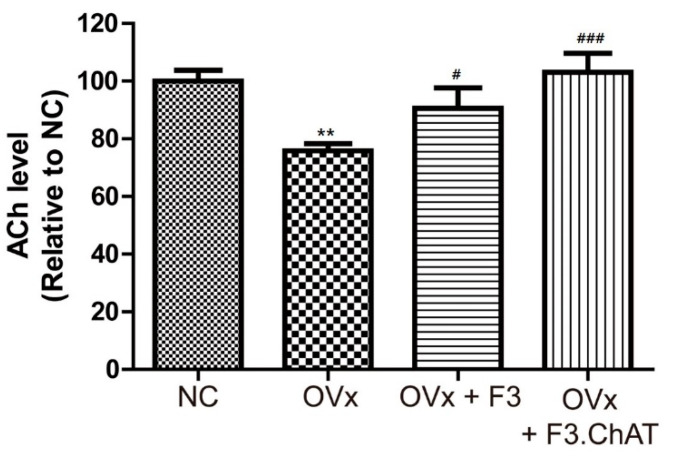
Acetylcholine (ACh) level in the CSF of ovariectomized rat four weeks after transplantation with F3 or F3.ChAT cells. ACh levels were analyzed using an ELISA kit. ** Significantly different from the normal control (*p* < 0.005). # Significantly different from the OVx (*p* < 0.05) group. ### Significantly different from the OVx (*p* < 0.05) group.

To confirm the relationship between memory deficits and decreases in ACh release, we analyzed the ACh concentration (Figure 7). The ACh concentration in the CSF of ovariectomized rats decreased to 80%; this was reversed by the transplantation of F3 and F3.ChAT cells. Notably, the transplantation of F3.ChAT cells resulted in an almost full reversal of the ACh concentration to the normal control level.

### 2.8. Transplantation of F3.ChAT Cells Enhances Expression of the Cholinergic System via Upregulation of NGF and BDNF

**Figure 8:**
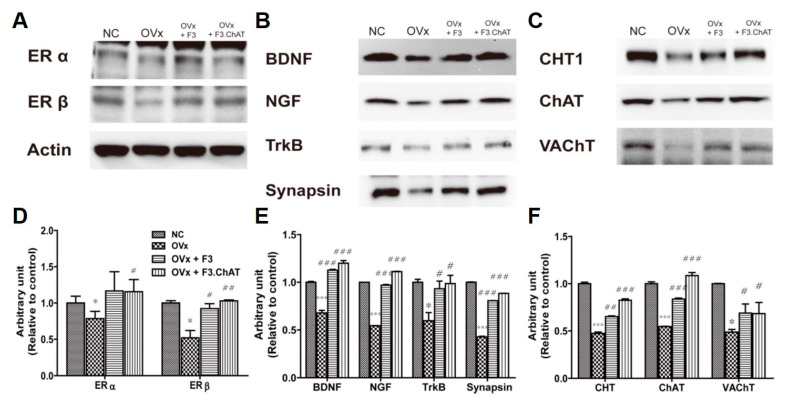
Expression of ER α and β (**A**), proteins related to the neurotrophin pathway (**B**), and the cholinergic system (**C**) in ovariectomized rats four weeks after transplantation of F3 or F3.ChAT cells. (**A**–**C**) Representative bands of ER α and β (**A**), proteins related to the neurotrophin pathway (**B**), and the cholinergic system (**C**). (**D**–**F**) The band densities were normalized to that of actin. * Significantly different from the normal control (*p* < 0.05). *** Significantly different from the normal control (*p* < 0.001). # Significantly different from the OVx (*p* < 0.05) group. ## Significantly different from the OVx (*p* < 0.05) group. ### Significantly different from the OVx (*p* < 0.05) group.

The expression of ERα and ERβ was markedly decreased in the brains of ovariectomized rats (Figure 8). The degenerative changes in estrogen receptors led to the decreased expression of the neurotrophin family proteins (NGF, BDNF, and Trk), resulting in decreased levels of cholinergic functional proteins such as CHT1, ChAT, and VAChT. Interestingly, transplantation of F3 and F3 ChAT cells restored the expression of the estrogen receptors, neurotrophin family proteins, and cholinergic functional proteins; F3.ChAT cells were more efficient than F3 cells.

**Figure 9:**
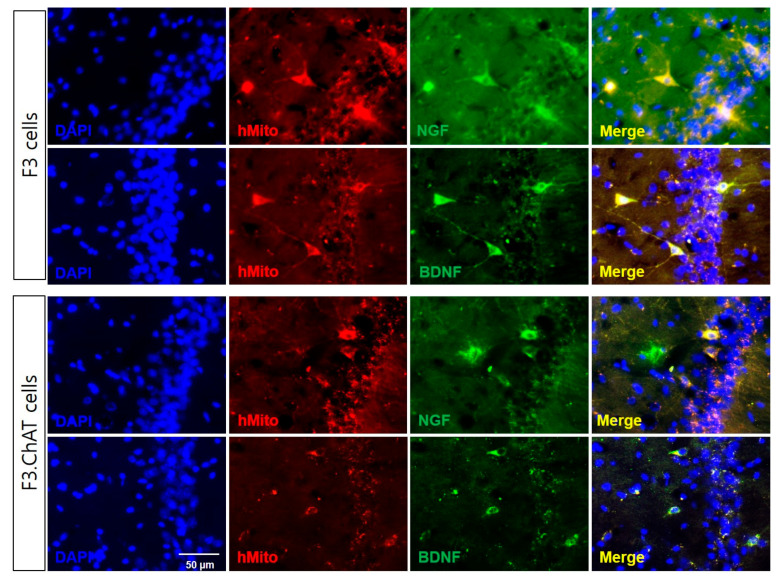
Representative immunohistochemical images indicating the expression of NGF and BDNF in F3 and F3.ChAT cells. Human mitochondria (hMito) was used as the transplanted F3 and F3.ChAT cell markers. Expression of NGF (Upper panel) and BDNF (Bottom panel) in the hippocampus of ovariectomized rat four weeks after transplantation of F3 and F3.ChAT cells were analyzed using double immunostating. Scale bar = 50 μm, magnification × 200.

The intravenous transplantation F3 and F3.ChAT cells were detected in the hippocampus of ovariectomized rats after four weeks (Figure 9). Double immunostaining indicated that the F3 and F3.ChAT cells (hMito-positive cells) expressed NGF and BDNF.

## 3. Discussion

In this study, we confirmed that estradiol had an influence on the cholinergic system; F3 and F3.ChAT cells improved cognitive function in ovariectomized rats via the neurotrophin signaling pathway. Estrogen receptors were co-localized with the neurotrophin receptor, and co-culture of primary cells and stem cells increased the concentrations of NGF and BDNF, resulting in the increased expression of CHT1, ChAT, and VAChT. In addition, transplantation of F3 and F3.ChAT cells in ovariectomized rats enhanced the levels of ACh, expression of estrogen receptors, NGF, and BDNF, and that of the cholinergic system, improving the memory dysfunction.

Estrogen deficiency during menopause induces diverse abnormal symptoms, including osteoporosis and weight gain [3,29]. In particular, it induces apoptosis, neuronal loss, and cognitive dysfunction in the hippocampus [30]. Therefore, several studies have focused on developing therapeutic strategies, such as hormone replacement, and understanding their mechanisms [31,32,33]. In this study, estradiol treatment increased the expression of the cholinergic system for ACh synthesis. The cholinergic system is an important site of action for estrogen in the brain. Estrogen facilitates cholinergic neurotransmission in the septal–hippocampal pathway, as evidenced by its ability to increase the activity and levels of ChAT [34]. Estrogen upregulates BDNF expression [7]. The increase in NGF and BDNF levels with exercise restores cognitive function in ovariectomized rats [35,36,37]. Therefore, estrogen receptors could modulate the expression of neurotrophin family proteins [38]. In this study, we confirmed that ERα and ERβ co-localized with neurotrophin family proteins. Therefore, estradiol treatment might upregulate the cholinergic system via the activation of the neurotrophin signaling pathway. Estrogen targets neurons in the basal forebrain, which expresses both NGF and ChAT [38,39,40].

Exogenous NGF and BDNF increased the expression of the cholinergic system. Stem cells facilitate the recovery of the damaged brain by the secretion of NGF and BDNF [13,14,41]. In this study, F3 and F3.ChAT cells secreted NGF and BDNF in the conditioned medium. When primary cells were co-cultured with F3 or F3.ChAT cells, the expression of endogenous BDNF, NGF, and their receptors increased. The PI3K/Akt signaling pathway involved in the neurotrophin pathway was upregulated [42]. These results are in line with those from earlier reports [43]. Exposure to neurotrophins increases ChAT activity and ACh release via Trk and calcium-dependent pathways [44,45]. Interestingly, co-culture with F3 and F3.ChAT cells decreased p75NTR levels; this could protect against apoptosis, because p75NTR signaling induces the apoptotic pathway [46,47]. The co-culture of primary cells with F3 and F3.ChAT increased the levels of CHT1, ChAT, and VAChT; the effect of F3.ChAT cells was higher than that of F3 cells. This could be attributed to the higher expression of NGF and BDNF in F3.ChAT cells compared to that in F3 cells.

Estradiol deficiency in ovariectomized rats decreases estrogen receptors, the uptake of high-affinity choline, ChAT activity, and ChAT mRNA levels [40,48]. NGF and BDNF mRNA levels were decreased after ovariectomy. In this study, we confirmed that the levels of the proteins associated with the cholinergic system (CHT, ChAT, and VAChT) and that of the neurotrophins (NGF and BDNF) decreased in the brain tissue of ovariectomized animals. In addition, the concentration of ACh in the CSF was reduced, resulting in memory deficits in behavioral tests. These effects could be reversed by exogenous estradiol, estradiol replacement therapy, and the upregulation of NGF and BDNF, either through exercise or the transplantation of F3 and F3.ChAT cells. In particular, F3.ChAT cells showed more potent effects than F3 cells in ovariectomized rats and Alzheimer’s animal models [23,49]. This is because F3.ChAT cells not only secrete more NGF and BDNF than F3 cells, but also produce more ACh by upregulating ChAT [14,49]. In this study, F3.ChAT cells showed a more potent effect than F3 cells in an in vitro experiment. However, in an in vivo experiment, F3.ChAT cells showed a mimic effect in a passive avoidance test and Morris water maze. It thought that most of stem cells are distributed to other peripheral organs and rapidly cleared after IV injection. After ICV injection, F3.ChAT cells showed more potent effect than F3 cells [14,23]. Interestingly, the levels of ERα and ERβ were also restored by transplantation of F3 and F3.ChAT cells. These could be a direct effect of replacing damaged cells by F3.ChAT cells, or an indirect effect of NGF and BDNF secreted from F3.ChAT cells. The estrogen receptors and BDNF were localized within the same cells, and estrogen significantly influenced the levels of BDNF mRNA and protein in a bidirectional manner within the adult rat hippocampus.

In general, hormone replacement therapy is performed for menopausal women. But it sometimes causes side effects such as breast tenderness, bloating, irritability and depression [50,51,52,53,54]. However, for memory deficiency, the up-regulation of NGF and BDNF using exercise or stem cells is thought to be a good alternative.

## 4. Materials and Methods

### 4.1. Primary Culture Cholinergic Neuron in Basal Forebrain

The septal region, including the basal forebrain, was obtained from BALB/c mice (Daehan Biolink, Eumseong, Korea) at embryonic day 15. Tissue was dissected in serum-free neurobasal medium (Gibco BRL, Grand Island, NY, USA) and treated with 0.03% trypsin in serum-free neurobasal medium for 3 min at 37 °C. After sieving through a 63 μm nylon mesh, cells were resuspended in serum-free neurobasal medium. The culture was initiated at 1 × 10^5^ cells per ml in 6 well plates coated with 10 μg/mL poly-D-lysine (PDL; Sigma-Aldrich, St. Louis, MO, USA) in neurobasal medium (Gibco) containing antibiotics (100 IU/mL penicillin and 100 ug/mL streptomycin) and 10% heat-inactivated fetal bovine serum (Gibco) at 37 °C in a 5% CO_2_/95% air atmosphere. To obtain glia-free neuronal cultures, cytosine arabinofuranoside (AraC, 1 μM, Sigma) was added to the cell culture media from days one to three to inhibit glial cell division.

To investigate the effect of estradiol on the cholinergic system, primary cells were washed twice with PBS, and then treated with 0, 5, 10, and 20 nM β-estradiol in culture medium (Sigma). After 24 h incubation, the cells were subjected to real-time PCR and western blotting analysis.

### 4.2. F3.ChAT Cell Culture

The establishment of F3 and F3.ChAT neural stem cell lines was described previously [14,49]. F3 (human neural stem cells) and F3.ChAT (ChAT overexpressing F3 cells) were cultured in Dulbecco’s modified Eagle’s medium (DMEM; Biowest, Nuaille’, Cholet, France) containing antibiotics (100 IU/mL penicillin and 100 μg/mL streptomycin) and 10% heat-inactivated fetal bovine serum (Biowest) at 37 °C in a 5% CO_2_/95% air atmosphere. In all experiments, cells were grown until more than 90% confluence and subjected to no more than 20 passages.

To investigate the effect of NGF and BDNF on the cholinergic system, primary cells were washed twice with PBS and were co-cultured with F3 and F3.ChAT cells. F3 and F3.ChAT cells were seeded at 1 × 10^5^ cells per ml in the upper chambers of 6-well transwell plates (pore 0.4 μm). After 24 h incubation, the cells were subjected to real-time PCR and western blotting analysis.

### 4.3. Assessing BDNF and NGF Levels

To measure NGF and BDNF levels in the medium, both F3 and F3.ChAT cells were seeded at 1 × 10^6^ cells/mL in six wells. After a 24 h incubation, BDNF and NGF levels in the medium were determined using enzyme-linked immunosorbent assay (ELISA). Human BDNF and NGF ELISA kits (Abcam, Cambridge, UK) were used according to the manufacturer’s instructions. O.D values were measured using an ELISA plate reader (MX2; Molecular Devices, Sunnyvale, CA, USA). The experiments were performed in triplicate, and the mean values are presented.

### 4.4. Immunocytochemistry of Estrogen Receptors and Neurophin Family in Primary Cells

To analyze the localization of the estrogen receptor and the neurotrophin family proteins, the primary cells were incubated in PDL coated 6 well plates and fixed in 10% neutral buffered formalin for 5 min at room temperature. After blocking for 10 min, the cells were incubated with primary antibodies specific for estrogen receptor α and β (1:100; mouse monoclonal, Abcam, Cambridge, UK) for overnight at 4 °C, followed by incubation with Alexa Fluor 488-conjugated anti-mouse IgG (Molecular Probes, Eugene, OR, USA) for 1 h at room temperature. The cells were washed thrice with PBS; for double immunostaining, the cells were incubated with primary antibodies specific for NGF, BDNF, TRK, and p75NTR (1:100; rabbit polyclonal, Abcam, Cambridge, UK) for 3 h at room temperature, followed by incubation with Alexa Fluor 594-cojugated anti-rabbit IgG (Molecular Probes) for 1 h at room temperature. Cells were counterstained with 4,6-diamino-2-phenylindole (DAPI; Sigma-Aldrich, St. Louis, MO, USA) to stain the nuclei and viewed under a fluorescence microscope (EVOS FL Auto2 Cell imaging system; Thermo Fisher Scientific, Waltham, MA, USA).

### 4.5. Quantitative Real Time PCR Analysis

Total RNA was isolated from primary cells using TRIzol Reagent (Invitrogen, Carlsbad, CA, USA), according to the manufacturer’s instructions. Quantitative real-time PCR was performed as described previously [55]. Glyceraldehyde 3-phosphate dehydrogenase (GAPDH) was used as the internal standard to normalize the expression of the target transcripts. The details of primers used to amplify the choline transporter (CHT), ChAT, and vesicular acetylcholine transporter (VAChT) are included in Table 1. Triplicate data were analyzed in three independent assays using the comparative Ct method [55].

### 4.6. Animals

Twelve-week-old female Sprague-Dawley rats were purchased from Daehan Biolink (Eumseong, Korea). Rats were housed in a controlled room with a constant temperature (23 ± 3 °C), relative humidity (50 ± 10%), and a 12-h light/dark cycle. Rats were fed a standard rodent diet and purified water ad libitum. All experimental procedures were approved and carried out in accordance with the Institutional Animal Care and Use Committee of the Korea National University of Education, Korea (#KNUE-202008-001-02).

### 4.7. Ovariectomy and Stem Cell Transplantation

Following the acclimation to the laboratory environment, the 12-week-old rats underwent bilateral ovariectomy, as previously described [6]. The rats were subjected to systemic anesthesia with ether and sterilized (10% povidone-iodine scrub followed by 70% alcohol wipe) before hair removal and treatment. A 1 cm incision was made in the center of the dorsal side of the experimental animal; the ovaries were ligated with suture threads, and ovarian resection was performed on both sides. Antibiotics (cafazolin 50 mg/kg) were administered through muscular injection.

Three weeks after recovery from surgery, 3 animals (normal and OVX rats) were subjected to passive avoidance and water maze tests to assess memory deficits. Other rats were randomly assigned to the following groups: normal group (NC; *n* = 10), OVX group (OVX; *n* = 10), OVX + F3 cells group (OVX-F3), and OVX + F3.ChAT cells group (OVX-F3.ChAT; *n* = 10). F3 or F3∙ChAT cells (1 × 10^6^ cells/rat) were transplanted into rats via tail vein injection.

### 4.8. Passive Avoidance Performance

Passive avoidance performance was assessed using a shuttle box (Med Associates, St. Albans, VT, USA) to evaluate memory acquisition and retention. The shuttle box apparatus consisted of light and dark compartments; a light chamber equipped with a lamp and a dark chamber with a steel-grid floor for electric shock. During the trials, an electric shock was delivered when the rats entered the dark compartment from the light room through a guillotine door. The latency time of remaining in a room with the light on following an electric shock (1 mA for 2 s) in a dark compartment was recorded. Four consecutive trials at 5-min intervals were performed with an electric shock when the rats entered the dark compartment. The endpoint was set to 300 s, denoting full memory acquisition.

### 4.9. Morris Water-Maze Performance

The Morris water-maze performance was assessed in a round water bath (180 cm in diameter; Panlab Technology, Barcelona, Spain) filled with water (27 cm in depth) maintained at 22 ± 2 °C to evaluate spatial memory. The bath was divided into four quadrants, and a hidden escape platform (10 cm in diameter, 25 cm in height) was submerged in the center of one quadrant, 2 cm below the water surface. The rats were subjected to four trials at 5 min intervals to find the hidden platform based on several cues external to the maze. The endpoint was set to 300 s if the animal failed to find the platform. The mean escape latency time, which was the time spent to escape from the platform during the trials, was recorded.

### 4.10. ACh Analysis in CSF

The rats were sacrificed at the end of the learning/memory testing, and CSF was collected to analyze ACh content [49]. The ACh concentration in the CSF was measured using the Amplex Red acetylcholine/acetylcholinesterase assay kit (Molecular Probes). In this assay, ACh is hydrolyzed by AChE to release choline, which is then oxidized by choline oxidase to betaine and H_2_O_2_. H_2_O_2_ interacts with Amplex Red (7-dihydroxyphenoxazine) in the presence of horseradish peroxidase to generate the highly fluorescent, resorufin. The resulting fluorescence was measured using a fluorescence microplate reader (MX2; Molecular Devices, Sunnyvale, CA, USA); excitation wavelength, 530–560 nm and emission wavelength, ~590 nm.

### 4.11. Western Blot Analysis in Primary Cells and Brain Tissues

Primary cells and brain tissue were homogenized in 10 volumes of RIPA buffer (Thermo Scientific, Waltham, MA, USA) containing protease inhibitors (Sigma-Aldrich, St. Louis, MO, USA) and phosphatase inhibitors (Sigma-Aldrich, St. Louis, MO, USA). Western blotting was performed as previously described. The membranes were immunoblotted with primary antibodies, followed by incubation with horseradish peroxidase-conjugated anti-rabbit and anti-mouse secondary antibodies (Jackson Laboratories). The antibodies used in this study are listed in Table 2. Band densities were measured using ImageJ software (NIH, Bethesda, MD, USA) and normalized to the density of the actin band.

### 4.12. Immunohistochemistry in Brain Sections

The brains of rats were perfusion-fixed with 10% paraformaldehyde solution and post-fixed in the same solution for 48 h, followed by cryoprotection in 30% sucrose for 72 h. Coronal cryosections of 30-μm thickness, 1.0 mm posterior to bregma were prepared and processed for double immunostaining of human mitochondria (hMito; for human cells), NGF, and BDNF using antibodies specific for hMito (1:200, mouse monoclonal, Chemicon), NGF (1:300, rabbit polyclonal, Abcam), and BDNF (1:300, rabbit polyclonal, Abcam). Brain sections were incubated with the primary antibodies overnight at 4 °C, followed by incubation with secondary antibodies conjugated with Alexa Fluor-488 or -594 (1:300, Molecular Probes) for 2 h at room temperature [6,7,8,9]. This was followed by staining with 4,6-diamidino-2-phenylindole (DAPI) for 30 min. All samples were evaluated immediately after staining and photographed using a fluorescence microscope (EVOS FL Auto2 Cell imaging system; Thermo Fisher Scientific, Waltham, MA, USA).

### 4.13. Statistical Analysis

Statistical comparisons between the groups were performed using one-way analysis of variance (ANOVA) followed by Tukey’s multiple comparison test. All analyses were conducted using Statistical Package for Social Sciences for Windows software (version 12.0; SPSS Inc., Chicago, IL, USA). Statistical significance was set at *p* < 0.05. All data are expressed as the mean ± SD.

## 5. Conclusions

Treatment with estradiol or with exogenous NGF and BDNF induced the cholinergic system. Transplantation of F3.ChAT cells restored memory dysfunction in ovariectomized rats via upregulation of NGF and BDNF. In addition, it restored the function of estrogen receptors. Upregulation of neurotrophins through stem cell transplantation or exercise improves abnormal symptoms in menopausal women, and this is a promising strategy for ameliorating the cognitive impairment caused by the loss or reduction of estradiol.

## Figures and Tables

**Table 1:** Sequences of the primers used in the current study.

Gene Name	Accession No.	Mouse Primer	
		Forward	Reverse
CHT	NM_022025.4	TTCCAGATTCAGGCAGTAGACG	GGGAGGGAAACTCCTATCTTGT
ChAT	NM_009891.2	TGGGTCTCTGAATACTGGCTGA	GGGCTAGAGTTGACTGGCAGG
VAChT	NM_021712.3	CCCTTTTGATGGCTGTGA	GGGCTAGGGTACTCATTAGA
GAPdH	XM_039103226	GTCGGTGTGAACGGATTTGG	CCACTTTGTCACAAGAGAAGGCA

**Table 2:** List of antibodies used in the current study.

Epitope	Company	Cat. Number	Dilution	2° Ab (IgG)
BDNF	Abcam	Ab226843	1:1000	Rb
NGF	Abcam	Ab6199	1:1000	Rb
TrkA	ThermoFisher	Pa5-98018	1:1000	Rb
TrkB	Abcam	Ab18987	1:2000	Rb
P75NTR	Abcam	Ab52987	1:1000	Rb
p-PI3K	Cell signaling	#4228	1:1000	Rb
p-AKT	Cell signaling	#9271	1:1000	Rb
AKT	Cell Signaling	#9272	1:1000	Rb
Synapsin	Abcam	Ab64581	1:1000	Rb
Actin	Cell Signal	#5125	1:1000	Rb
ER α	Abcam	Ab66102	1:100	Ms
ER β	Abcam	Ab288	1:100	Ms
CHT1	Abcam	Ab154186	1:1000	Rb
ChAT	Abcam	Ab178850	1:1000	Rb
VAChT	Synaptic systems	#139103	1:500	Rb

## Data Availability

All data generated from this study are contained within the article.
